# Systematic Review and Bioinformatic Analysis of microRNA Expression in Autism Spectrum Disorder Identifies Pathways Associated With Cancer, Metabolism, Cell Signaling, and Cell Adhesion

**DOI:** 10.3389/fpsyt.2021.630876

**Published:** 2021-10-21

**Authors:** Zhi-Xiong Huang, Yanhui Chen, Hong-Ru Guo, Guo-Feng Chen

**Affiliations:** Department of Pediatrics, Fujian Medical University Union Hospital, Fuzhou, China

**Keywords:** systematic review, microRNA, autism spectrum disorder, gene, signaling pathway, bioinformatic analysis

## Abstract

**Background:** Previous studies have identified differentially expressed microRNAs in autism spectrum disorder (ASD), however, results are discrepant. We aimed to systematically review this topic and perform bioinformatic analysis to identify genes and pathways associated with ASD miRNAs.

**Methods:** Following the Preferred Reporting Items for Systematic reviews and Meta-Analyses, we searched the Web of Science, PubMed, Embase, Scopus, and OVID databases to identify all studies comparing microRNA expressions between ASD persons and non-ASD controls on May 11, 2020. We obtained ASD miRNA targets validated by experimental assays from miRTarBase and performed pathway enrichment analysis using Metascape and DIANA-miRPath v3. 0.

**Results:** Thirty-four studies were included in the systematic review. Among 285 altered miRNAs reported in these studies, 15 were consistently upregulated, 14 were consistently downregulated, and 39 were inconsistently dysregulated. The most frequently altered miRNAs including miR-23a-3p, miR-106b-5p, miR-146a-5p, miR-7-5p, miR-27a-3p, miR-181b-5p, miR-486-3p, and miR-451a. Subgroup analysis of tissues showed that miR-146a-5p, miR-155-5p, miR-1277-3p, miR-21-3p, miR-106b-5p, and miR-451a were consistently upregulated in brain tissues, while miR-4742-3p was consistently downregulated; miR-23b-3p, miR-483-5p, and miR-23a-3p were consistently upregulated in blood samples, while miR-15a-5p, miR-193a-5p, miR-20a-5p, miR-574-3p, miR-92a-3p, miR-3135a, and miR-103a-3p were consistently downregulated; miR-7-5p was consistently upregulated in saliva, miR-23a-3p and miR-32-5p were consistently downregulated. The altered ASD miRNAs identified in at least two independent studies were validated to target many autism risk genes. *TNRC6B, PTEN, AGO1, SKI*, and *SMAD4* were the most frequent targets, and miR-92a-3p had the most target autism risk genes. Pathway enrichment analysis showed that ASD miRNAs are significantly involved in pathways associated with cancer, metabolism (notably Steroid biosynthesis, Fatty acid metabolism, Fatty acid biosynthesis, Lysine degradation, Biotin metabolism), cell cycle, cell signaling (especially Hippo, FoxO, TGF-beta, p53, Thyroid hormone, and Estrogen signaling pathway), adherens junction, extracellular matrix-receptor interaction, and Prion diseases.

**Conclusions:** Altered miRNAs in ASD target autism risk genes and are involved in various ASD-related pathways, some of which are understudied and require further investigation.

## Introduction

Autism spectrum disorder (ASD) is a category of clinically and genetically heterogeneous neurodevelopmental disorder characterized by impaired social function and repetitive, restricted behaviors (1). Genetics plays a significant role in the cause of ASD. Hundreds of different genetic loci, including non-coding mutations (2), single-nucleotide variants, chromosome abnormalities, and copy number variations have been associated with ASD (3, 4). However, many different variants share common biological pathways (5). Thus, identifying converging biological pathways and molecular mechanisms responsible for this disorder has much translational and clinical value (5).

MicroRNAs (miRNAs) are a class of small, non-coding RNAs with the main functions of regulating mRNA destabilization and modifying protein levels (6). One miRNA can target up to hundreds of different mRNAs, and a single mRNA may be regulated by many miRNAs. Thus, miRNAs–mRNAs form complicated gene regulatory networks and participate in various biological functions (7), including brain development and function (8). By regulating local gene expression, miRNAs can control cell fate determination, neurogenesis, cell migration, neuronal polarization, synapse development, and synaptic plasticity (9). In neurodevelopmental disorders, miRNAs are often dysregulated, indicating that miRNAs play an important part in the etiology and/or maintenance of neurological disorders (9).

Since the first study performed by Abu-Elneel and his colleagues in 2008 (10), miRNA expression profiling in ASD has been performed in brain tissue samples (10–18), serum (19–23), blood (24–30), peripheral blood monocytes (31–33), lymphoblast cell lines (34–37), saliva (38–40), reprogrammed induced pluripotent stem cell-derived neurons (41), olfactory mucosal stem cells, and skin fibroblasts (42) in studies comparing ASD persons and controls. There were a significant number of miRNAs in ASD samples and control with different expressions; however, many discrepancies among studies exist. The discrepancies may be due to differences in tissue types, genetic and environmental origin of the tissue sources, RNA extraction methods, miRNA detection, and validation methods (such as microarray, miRNA-seq, and RT-qPCR), data normalization methods, identification of new miRNAs, and miRNAs' annotation changes in the miRbase database, which stores information about individual microRNAs since 2002 (43). The biological function of these microRNAs in ASD pathogenesis remains little known, and there remains a question as to which miRNAs may be significant specific signatures as personalized biomarkers or therapeutic targets.

Until now, the literature reviews of ASD miRNAs were primarily narrative, and conclusive results were not available for comprehensive functional analysis (44). Therefore, an updated systematic review is needed. The objective of this research was to systematically review the literature to assess which microRNAs were altered in children and adults with ASD when compared to non-ASD controls from case-control studies and standardize them to miRBase version 22.1 (43). Furthermore, validated gene targets of these miRNAs were obtained and pathway enrichment analysis was used to assess the physiological impact of miRNA dysregulation in ASD pathology. Our study may clarify the ambiguities and contradictions in this research field and promote future further studies to better understand microRNAs' function in ASD. This may also contribute to the use of microRNAs as potential personalized biomarkers and therapeutic targets.

## Materials and Methods

### Criteria for Considering Studies for This Review

#### Types of Participants

We included children and adults with ASD diagnosed by an established classification system or clinical assessment, including individuals with autistic disorder, Asperger's disorder, and pervasive developmental disorder–not otherwise specified (PDD-NOS). Participants with comorbidities were not excluded.

#### Types of Exposures

The exposure was ASD diagnosis.

#### Types of Control

The control participants were non-ASD individuals.

#### Types of Outcome Measures

The outcome measure was microRNA expression level.

#### Types of Studies

Original research case-control studies written in English were eligible for this review. Literature reviews, non-human studies, comments, opinion articles, expert opinions, letters, news reports, hypotheses, conference summaries, book sections, patent descriptions, same study reports, non-ASD or non-microRNA expression studies, and studies that are not case-control were excluded.

### Database Search Strategies for Identification of Studies

We conducted a systematic literature search in Web of Science, PubMed, Embase, Scopus, and OVID databases without initial date restriction up to and including May 11, 2020. The search criteria in five databases are provided in Supplementary Material 1. No language restriction was applied. The search yielded 2,718 references.

### Selection of Studies

**Figure 1:**
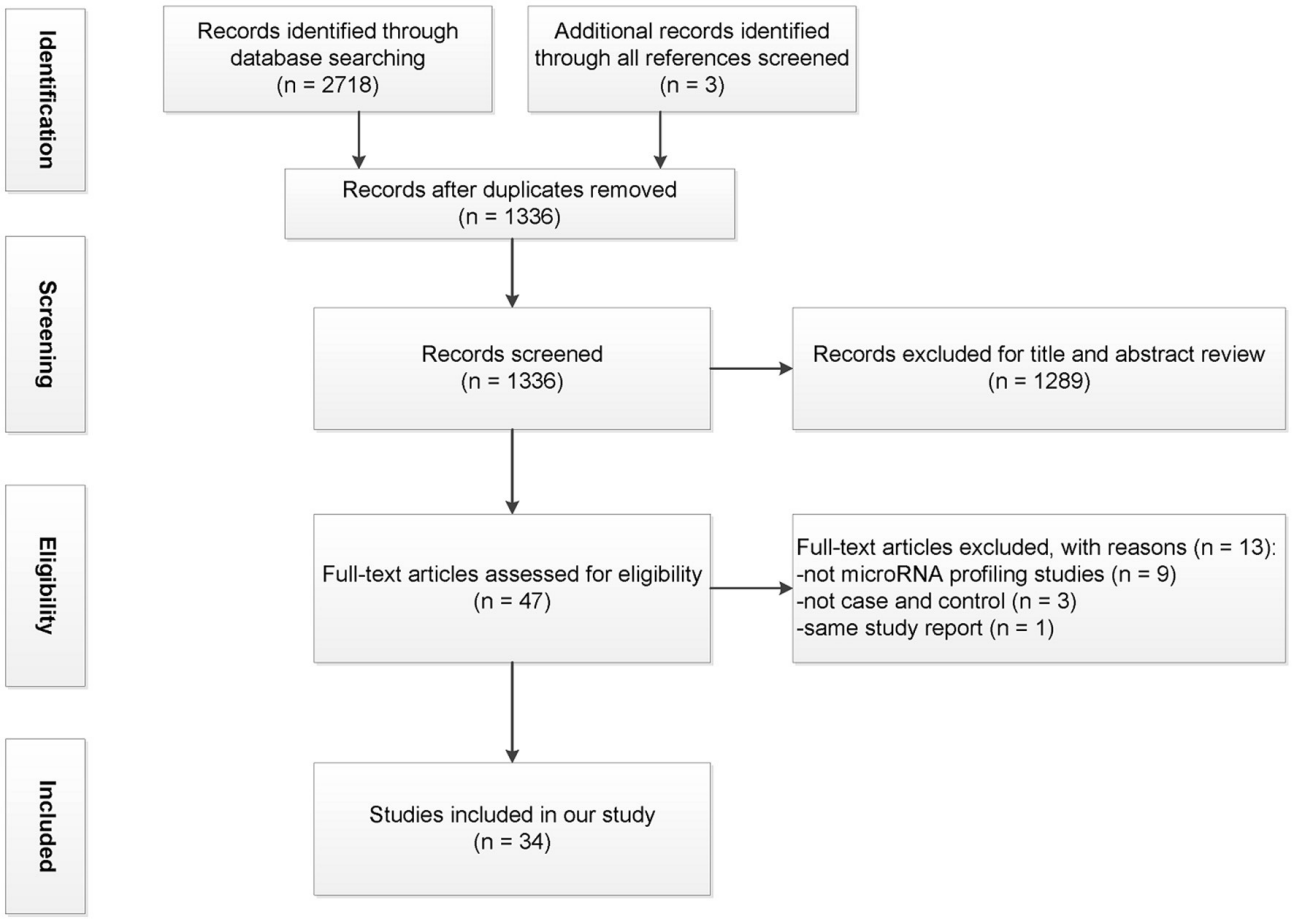
Flowchart illustrating the process of study selection.

All references were managed in the EndNote X9 software (Thomson Reuters, New York, NY, USA). Initially, duplicate references were removed. Two reviewers (ZXH and GFC) independently screened titles and abstracts based on the inclusion and exclusion criteria. Lists were compared, and a consensus was reached through discussion or with a third reviewer in case of disagreement. This systematic review followed the Preferred Reporting Items for Systematic reviews and Meta-Analyses (PRISMA) statements (Figure 1; Supplementary Material 2), with some modifications (45).

### Data Collection

Two reviewers (ZXH and HRG) independently collected the following data items from the full text and supplementary data of each included study: first author, year of publication, country of study, tissue types, diagnostic measure, sample sizes, the age of cases and controls, the gender of cases and controls, miRNA expression assay type, lists of upregulated and downregulated miRNAs with statistically significant expression changes, and differential expression criteria. Lists were compared, and a consensus was reached through discussion or with a third reviewer (GFC) in case of disagreement. All miRNA names from different studies were standardized to miRBase version 22.1 (43). Pre-miRNAs, viral miRNAs, and non-miRNAs were not used in the bioinformatic analysis.

### Enrichment for ASD Risk Genes Among Validated Targets for Each Overlapping miRNAs in ASD

To explore the experimentally validated target genes of each microRNAs that were dysregulated in at least two independent studies (ASD-miRNAs), we obtained ASD-miRNAs targets that had been experimentally verified using reporter assays, western blots, microarrays, or next-generation sequencing studies, among other methods, from the miRTarBase database (Supplementary Material 3) (46). We did not perform further filtering based on strength of evidence. Next, we systematically evaluated and compared whether targets of the differentially expressed miRNAs are enriched for ASD risk genes (Supplementary Material 4) from the Simons Foundation Autism Research Initiative (SFARI, https://gene.sfari.org/database/human-gene/) (47) and AutDB database (http://autism.mindspec.org/autdb/HG_Home.do) (48), which are based on candidate gene studies, common variant association, genetic syndromes, and copy number variation.

### Subgroup Analysis of Tissues and Pathway Enrichment Analysis of Validated Targets for Each ASD-miRNAs

To further explore the associated biological pathways of ASD-miRNAs in specific tissues, firstly the web-based software portal Metascape was used to conduct Kyoto Encyclopedia of Genes and Genomes (KEGG) and Reactome pathways analysis of validated targets from miRTarbase for each of ASD-miRNAs (49), with a background set of genes that are expressed in the brain, salivary glands, blood, and immune cells (Supplementary Material 5, data available from v20.1.proteinatlas.org) (50) for miRNA detected in these tissues. There are 16,227 genes in the brain (data available from https://www.proteinatlas.org/humanproteome/brain/human+brain), 15,218 in the salivary glands (data available from https://www.proteinatlas.org/humanproteome/tissue/salivary+gland), and 14,812 in “blood and immune cells” (data available from https://www.proteinatlas.org/humanproteome/celltype/blood+26+immune+cells). Studies using brain tissue, reprogrammed induced pluripotent stem cell-derived neurons, or olfactory mucosal stem cells were classified as “brain” tissue. Studies using serum, blood, lymphoblast cell lines, or peripheral blood mononuclear cells were classified as “blood or immune cell” tissue. Studies using saliva were classified as “salivary gland.” Terms with *p*-value < 0.01, a minimum count of 3, and enrichment factor of >1.5 were considered as significant (using hypergeometric test and Benjamini–Hochberg *p*-value correction).

### Explore KEGG Pathways That Are Commonly Targeted by Multiple miRNAs

Moreover, to investigate KEGG pathways that are simultaneously controlled by multiple ASD-miRNAs, dysregulated ASD-miRNAs in the brain, or consistently upregulated and downregulated ASD-miRNAs in blood or saliva, were inputted into the online software DIANA-miRPath v3.0 (51), respectively. The KEGG analysis was used, and the human species was selected. In the optional gene filter menu, genes that are expressed in the brain, salivary glands, blood, and immune cells (data available from v20.1.proteinatlas.org, http://www.proteinatlas.org) (50) were uploaded respectively. The interactions dataset selected for all microRNAs was TarBase v7.0, based on previous positive and negative experiments. In the advanced statistics options, the Fisher's Exact Test (Hypergeometric Distribution), Benjamini–Hochberg's False Discovery Rate (FDR) correction, and more conservative statistics (DAVID's EASE score) with a *p*-value threshold of 0.05 were selected. After inputting miRNAs into the software, “Pathways union” was selected to identify pathways containing more than one associated miRNA, which can give meta-analysis statistics for the assessment of combined miRNA action.

### Quality Assessment of Selected Studies

We assessed the methodological quality of the included studies using the Newcastle-Ottawa Scale (NOS) for case-control studies (52). The scale includes three categories: selection, comparability, and exposure. A higher total quality score indicates better study quality, and the highest quality score is 10 (Supplementary Material 6).

## Results

### Selection and Characteristics of Included Independent Studies

Using Web of Science, PubMed, Embase, Scopus, and OVID databases, a total of 2,718 studies were identified; another three articles were added from the review citation. Among them, 1,385 were duplicates; a total of 1,336 articles were included in the title and abstract screening after which 48 articles remained. After evaluating the full text, 14 studies were excluded. Figure 1 displays the PRISMA flowchart for the study selection process.

Details of the characteristics of the selected studies are shown in Supplementary Material 6. The year of publication was from 2008 to 2020. In total, this review pooled results from over 1,000 subjects with ASD and almost 1,000 controls. Five studies included only male participants (10, 18, 29, 35, 41). One study included only female participants (53). In all, there are more males than females with ASD (773:192). Participants' ages ranged from 2 to 81 years. In all 34 included studies, the participants had ASD diagnoses. The Autism Diagnostic Interview-Revised (ADI-R) was the most used diagnostic instrument among the studies, followed by the Diagnostic and Statistical Manual of Mental Disorders, 5th Edition (DSM-5), Autism Diagnostic Interview-Revised (ADOS), Diagnostic and Statistical Manual of Mental Disorders, 4th Edition, Text Revision (DSM-IV-TR), and Diagnostic and Statistical Manual of Mental Disorders, 4th Edition (DSM-IV). Six studies did not provide the ASD diagnostic criteria but did report that the participants had ASD, including autistic disorder, Asperger's disorder, and pervasive developmental disorder–not otherwise specified (PDD-NOS) (10, 14, 18, 33, 35, 53); thus, they are included in this study. Ten of these studies involved brain tissue (10–18, 53), 15 involved peripheral blood (19–33), four employed lymphoblastoid cell lines (34–37), three examined saliva (38–40), 1 examined reprogrammed induced pluripotent stem cell-derived neurons (41), and 1 examined olfactory mucosal stem cells and skin fibroblasts (42). Variation in these studies is due to differences in the patients investigated, the tissue examined, microRNA profiling and analysis methods, the number of samples, and the statistical analysis approach used.

### Differentially Expressed miRNAs

In the 34 selected miRNA expression profiling studies, 285 differentially expressed mature miRNAs were reported that compared over 1,000 subjects with ASD and almost 1,000 controls (Supplementary Material 6). Of the 68 differentially expressed miRNAs identified in at least two studies (ASD-miRNAs), 29 miRNAs had a consistent direction, 15 upregulated and 14 downregulated (Table 1), and 39 inconsistently dysregulated (Table 2). Among them, miR-23a-3p, miR-106b-5p, miR-146a-5p, miR-7-5p, miR-27a-3p, miR-451a, miR-181b-5p, and miR-486-3p are the most frequently reported (Tables 1, 2). Certain microRNAs changed in different ways depending on gender; for example, miR-148b-3p was shown to be downregulated solely in male ASD participants (Tables 1, 2; Supplementary Material 6).

**Table 1:** Consistently dysregulated miRNAs reported in at least two expression profiling studies.

**miRNA**	**Chromosome location**	**Studies/References**	**No. of ASD samples (male: female)**	**Male: female ratio of ASD samples**	**No. of autism risk genes targeted by the miRNA^a^**
**Upregulated**					
miR-146a-5p	5	(11, 17, 34, 42, 54)	N/A	N/A	24
miR-155-5p	21	(11, 13, 18)	63:12	5–6: 1	77
miR-106a-5p	X	(10, 22)	27:2	13–14: 1	49
miR-10a-5p	17	(13, 36)	58:17	3–4: 1	43
miR-335-3p	7	(13, 38)	64:15	4–5: 1	35
miR-142-3p	17	(11, 28)	34:8	4–5: 1	34
miR-23b-3p	9	(34, 35)	14:3	4–5: 1	28
miR-107	10	(13, 35)	56:10	5–6: 1	27
miR-223-3p	X	(13, 37)	45:10	4–5: 1	11
miR-146b-5p	10	(10, 34)	16:3	5–6:1	13
miR-483-5p	11	(26, 32)	105:20	5–6:1	8
miR-21-3p	17	(11, 13)	55:12	4–5:1	6
miR-191-5p	3	(35, 38)	30:5	6:1	5
miR-1277-3p	X	(13, 54)	45:11	4–5:1	1
miR-494	14	(11, 26)	27:5	5–6:1	N/A
**Downregulated**					
miR-15a-5p	13	(10, 26, 28)	54:9	6:1	73
let-7a-5p	9, 11, 22	(11, 26, 39)	188:31	6–7:1	48
miR-193a-5p	17	(29, 32, 39)	256:43	5–6:1	3
miR-92a-3p	X, 13	(26, 34)	20:6	3–4:1	118
miR-15b-5p	3	(10, 26)	30:3	10:1	91
miR-20a-5p	13	(26, 32)	105:20	5–6:1	71
miR-148b-3p	12	(10, 35)	24:0	24:0	45
miR-211-5p	15	(11, 35)	21:2	10–11:1	44
miR-4742-3p	1	(12, 15)	10:10	1:1	11
miR-148a-5p	7	(32, 39)	249:43	5–6:1	10
miR-151a-3p	8	(19, 39)	209:33	6–7:1	6
miR-3135a	3	(23, 28)	48:12	4:1	5
miR-574-3p	4	(26, 32)	105:20	5–6:1	3
miR-3687	21, 22	(13, 28)	69:16	4–5:1	3

a *Based on validated target genes in miRTarbase, N/A: not available*.

**Table 2:** Inconsistently dysregulated miRNAs reported in at least two expression profiling studies.

**miRNA**	**Genome location^a^**	**Studies/References**		**N ASD (male: female) Up**	**Male: female ratio of N ASD Up**	**N ASD (male: female) Down**	**Male: female ratio of N ASD Down**
		**Up**	**Down**				
miR-23a-3p	19	(13, 34, 35, 54)	(10, 38, 40)	59:14	4–5:1	57:19	3:1
miR-106b-5p	7	(10, 19, 28, 35, 54)	(35)	96:14	6–7:1	11:0	11:0
miR-7-5p	9, 15, 19	(11, 38, 40)	(10, 32)	54:21	2–3:1	101:17	5–6:1
miR-181b-5p	1, 9	(33, 36)	(19, 33)	39:18	2–3:1	74:18	4–5:1
miR-27a-3p	19	(19)	(10, 29, 38)	48:7	6–7:1	39:5	7–8:1
miR-486-3p	8	(21, 36)	(24, 28)	31:9	3–4:1	48:12	4:1
miR-451a	17	(11, 16, 35)	(26)	29:3	9–10:1	17:3	5–6:1
miR-93-5p	7	(35)	(10, 32)	11:0	11:0	101:17	5–6:1
miR-19b-3p	13, X	(11, 19)	(26)	58:9	6–7:1	17:3	5–6:1
miR-195-5p	17	(19, 35)	(26)	59:7	8–9:1	17:3	5–6:1
miR-21-5p	17	(11)	(10, 54)	10:2	5:1	13:1	13:1
miR-103a-3p	5, 20	(54)	(26, 32)	0:1	0:1	105:20	5–6:1
miR-32-5p	9	(37)	(38, 40)	N/A	N/A	44:19	2–3:1
miR-145-5p	5	(29)	(28, 32)	7:0	7:0	112:23	4–5:1
miR-619-5p	12	(28, 54)	(13)	24:7	3–4:1	45:10	4–5:1
miR-132-3p	17	(34)	(10, 35)	3:3	1–1:1	24:0	24:0
miR-140-3p	16	(20, 38)	(40)	41:13	3–4:1	25:14	1–2:1
miR-199a-5p	1, 19	(29, 36)	(28)	20:7	2–3:1	24:6	4:1
miR-328-3p	16	(30)	(23, 28)	18:12	1–2:1	48:12	4:1
miR-484	16	(13)	(10)	45:10	4–5:1	13:0	13:0
miR-193b-3p	16	(10)	(28)	13:0	13:0	24:6	4:1
miR-320a	8	(10)	(19)	13:0	13:0	48:7	6–7:1
miR-186-5p	1	(35)	(37)	11:0	11:0	N/A	N/A
miR-424-5p	X	(13)	(28)	45:10	4–5:1	24:6	4:1
miR-221-3p	X	(13)	(42)	45:10	4–5:1	N/A	N/A
miR-144-3p	17	(11)	(30)	10:2	5:1	18:12	1–2:1
miR-940	16	(13)	(26)	45:10	4–5:1	17:3	5–6:1
miR-223-5p	X	(37)	(32)	N/A	N/A	88:17	5–6:1
miR-423-5p	17	(32)	(37)	88:17	5–6:1	N/A	N/A
miR-34c-5p	11	(29)	(37)	7:0	7:0	N/A	N/A
miR-363-3p	X	(13)	(34)	45:10	4–5:1	3:3	1:1
miR-663a	20	(34)	(19)	3:3	1:1	48:7	6–7:1
miR-338-5p	17	(11)	(15)	10:2	5:1	5:5	1:1
miR-199b-5p	9	(36)	(35)	13:7	1–2:1	11:0	11:0
miR-379-5p	14	(11)	(32)	10:2	5:1	88:17	5–6:1
miR-204-3p	9	(54)	(13)	0:1	0:1	45:10	4–5:1
miR-628-5p	15	(38)	(40)	19:5	3–4:1	25:14	1–2:1
miR-127-3p	14	(38)	(54)	19:5	3–4:1	0:1	0:1
miR-874-3p	5	(13)	(30)	45:10	4–5:1	18:12	1–2:1

a *Chromosome location, N/A: not available*.

Subgroup analysis of tissues showed that in brain samples, six miRNAs (miR-146a-5p, miR-155-5p, miR-1277-3p, miR-21-3p, miR-106b-5p, and miR-451a) were consistently upregulated, 1 miRNA (miR-4742-3p) was consistently downregulated, and seven miRNAs were inconsistently dysregulated (Supplementary Material 6). In blood and immune cell samples, four miRNAs (miR-146a-5p, miR-23b-3p, miR-483-5p, and miR-23a-3p) were consistently upregulated, seven miRNAs (miR-15a-5p, miR-193a-5p, miR-20a-5p, miR-574-3p, miR-92a-3p, miR-3135a, and miR-103a-3p) were consistently downregulated, and 19 miRNAs were inconsistently dysregulated (Supplementary Material 6). In saliva samples, one miRNA (miR-7-5p) was consistently upregulated, two miRNAs (miR-23a-3p and miR-32-5p) were consistently downregulated, and two miRNAs were inconsistently dysregulated (Supplementary Material 6).

### Validated Gene Targets of Each Dysregulated microRNAs Overlap With ASD Risk Genes

Compared to experimentally validated microRNA-targets based on miRTarBase, each ASD-miRNA targets many genes. miR-92a-3p, miR-15b-5p, miR-93-5p, and miR-155-5p are among microRNAs that have the greatest number of validated ASD risk gene targets (Table 1, Supplementary Materials 7–9). The most frequently targeted ASD candidate genes were *TNRC6B, PTEN, AGO1, AGO2, SKI*, and *SMAD4* (Supplementary Materials 7–9).

### Subgroup Analysis of Tissues and ASD-miRNAs Associated KEGG and Reactome Pathways

**Figure 2:**
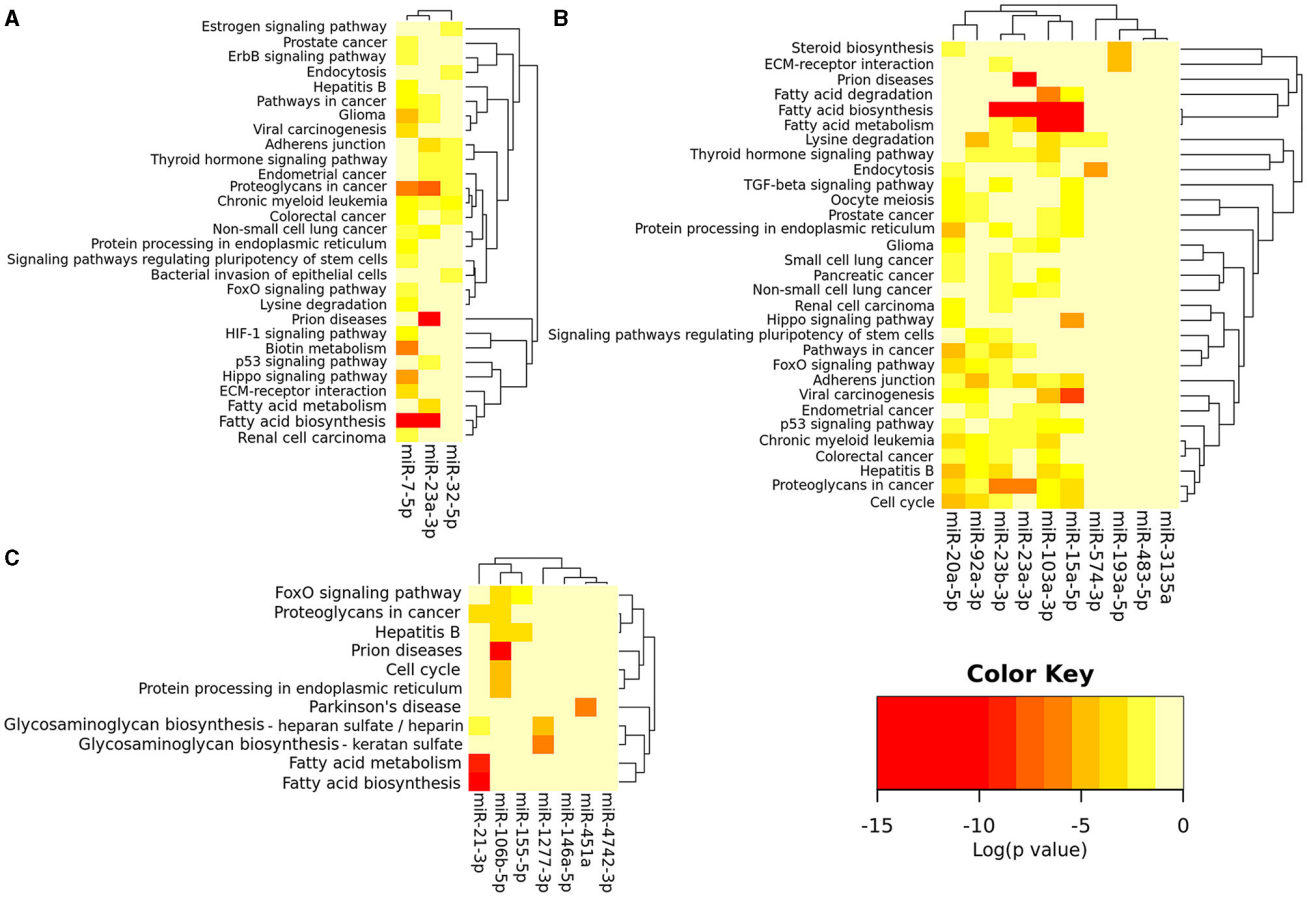
miRNAs vs. pathways heatmap (Clustering based on significance levels) of the consistently dysregulated ASD-miRNAs in blood and immune cells **(A)**, saliva **(B)**, and brain tissue **(C)**. The heatmap was created from the DIANA mirPath v 3.0. Darker shades represent more significant. Dendrograms depicts hierarchical clustering results for miRNAs and pathways, respectively. The miRNA axis represents the clustered miRNAs with similar pathway targeting patterns. A similar clustering is shown on the pathway axis. Interactions dataset: TarBase v7.0; Analysis: pathways union; *p*-value threshold: 0.05; FDR Correction, Conservative Statistics.

Using the Metascape, a diverse range of KEGG and Reactome pathways were significantly enriched for validated gene targets of each dysregulated microRNAs (Supplementary Materials 7–9). Furthermore, using the software DIANA-mirPath v.3, bioinformatics analysis revealed that among the 7, 11, and 3 consistently dysregulated ASD-miRNAs in the brain, blood, and saliva respectively, there were various commonly targeted pathways (Figures 2A–C). Enriched KEGG pathways were most significantly associated with cancer, metabolism (notably steroid biosynthesis, fatty acid metabolism, fatty acid biosynthesis, lysine degradation, biotin metabolism), cell cycle, cell signaling (especially Hippo, FoxO, TGF (transforming growth factor)-beta, p53, thyroid hormone, and estrogen signaling pathway), adherens junction, extracellular matrix–receptor interaction, prion diseases, etc., (Figures 2A–C).

## Discussion

In this report, we systematically reviewed the ASD-related miRNAs from 34 independent profiling studies. Up until now, more than 285 mature microRNAs have been identified (Supplementary Material 6), of which 68 had altered levels in at least two independent studies comparing autistic persons with controls (Tables 1, 2). The most frequently altered miRNAs in all studies include miR-23a-3p, miR-106b-5p, miR-146a-5p, miR-7-5p, and miR-27a-3p. MiRNAs that were dysregulated in at least two studies were validated to target a number of ASD risk genes. Among them, *TNRC6B, PTEN, AGO1, AGO2, SKI*, and *SMAD4* were the most frequent (Supplementary Materials 7–9), and miR-92a-3p has the most target ASD risk genes (Table 1, Supplementary Material 8). Bioinformatic analysis showed that validated targets of each microRNA were enriched in various KEGG and Reactome pathways. Moreover, tissue subgroup enrichment analysis by using mirPath v.3 showed that various commonly targeted pathways were identified for the 7, 11, and 3 consistently dysregulated ASD-miRNAs in brain, blood, and saliva, respectively. Our study clarified miRNAs involved in ASD and explored their target genes and significantly associated pathways, which are the focus of our discussion next.

miR-23a-3p and miR-27a-3p belong to the same cluster and participate in several ASD-related pathways, such as lysine degradation and fatty acid metabolism (Supplementary Materials 7–9; Figures 2A,B). Our results showed that miR-23a-3p targets many ASD risk genes, including *PTEN, TSC1*, and *KDM3B* (Supplementary Material 7). miR-23a plays a crucial role in neurogenesis (9). For example, miR-23a-3p controls oligodendrocyte differentiation and myelin formation by targeting *PTEN* and manipulating PTEN/PI3K/Akt/mTOR pathway (55). It also directly targets *TSC1*, which is crucial in the mTOR signaling pathway associated with synaptic protein defects in autistic persons (54). miR-27a-3p targets many ASD risk genes, including *IGF1, KMT2A, KMT2C, KMT5B*, and *IL6* (Supplementary Material 8). For example, miR-27a-3p targets insulin-like growth factor-1 (*IGF-1*), which binds to the IGF-1 receptor and activates PI3K and MAPK pathways and various downstream signaling pathways, including mTOR, p53, and FoxO signaling pathway. Insulin-like growth factor-1 plays an important role in brain development (56); it has shown a potential therapeutic role for ASD *in vitro* (57) and in individuals with ASD (58) and Rett syndrome (59). *KMT2A, KMT2C, KMT5B*, and *NSD1* all participate in post-translational chromatin modification such as histone lysine methylation and are thus linked to transcriptional activation or repression (60). These genes are also involved in lysine degradation and play an important role in both normal human development and developmental disorders, including ASD (61). A recent study showed that miR-27a-3p has protective effects on the blood–brain barrier, brain injury, and hippocampal neuron injury (62). Therefore, its dysregulation may result in neurologic dysfunction. miR-23a-3p and miR-27a-3p are dysregulated not only in brain samples (10) but also in the saliva of autistic persons (38). Thus, they may be potential biomarkers of ASD.

hsa-miR-106b-5p and hsa-miR-93-5p belong to the microRNA-25-93-106b cluster; they are vital for “neural stem/progenitor cell proliferation and neuronal differentiation” and are involved in various psychiatric or neurologic disorders (63, 64). They both target many ASD risk genes including *MECP2, PTEN*, and *SMAD4*, and they are involved in various pathways, including adherens junction, circadian clock, long-term depression, mTOR, and estrogen signaling pathway (Supplementary Materials 7, 8). Methyl CpG binding protein 2 (*MECP2*) is abundantly expressed in neurons and is essential for neuronal function and development (65). *MECP2* under-expression and overexpression have been associated with Rett syndrome (3, 66) and *MECP2* duplication syndrome, respectively, and many neurologic disorders including ASD (3, 67). Besides, many cases of Rett syndrome and *MECP2* duplication syndrome fit the diagnostic criteria for ASD (3, 68). As a multifunctional protein, MECP2 affects various metabolites (including glutamate and catecholamine catabolites), genes (including *BDNF*), and pathways (including AKT/mTOR and neurotrophin signaling pathway). Therefore, more research is needed to investigate the target genes and pathways of hsa-miR-106b-5p and hsa-miR-93-5p to better understand neurologic disorders including ASD and Rett syndrome.

Notably, miR-146a-5p (11, 17) and miR-155-5p (11, 13, 18) were both consistently upregulated in brain tissues of ASD persons (Supplementary Material 6), and they play a critical role in regulating immune response (69) and neuroinflammation (70). In line with this, our results showed that they target immune/inflammation-associated genes (including *IL6* and *SMAD4*) and pathways (such as NF-kappa B, TGF-beta, and Toll-like receptor signaling pathway) (Figure 2C; Supplementary Material 7). miR-146a-5p has also been consistently upregulated in lymphoblastoid cell line (34), skin fibroblasts (42), and olfactory mucosal stem cell (42) samples from people diagnosed with ASD. This may be an anti-inflammatory compensatory reaction since miR-146a-5p primarily acts as a negative inflammation regulator (70). However, evidence showed that miR-146a-5p dysregulation can affect neuronal development and differentiation and may thus lead to ASD (17, 42, 71).

miR-7-5p is abundant in neurons and neuroendocrine organs and is a prototypical neuroendocrine miRNA. It regulates many genes in the brain (Supplementary Material 7) (72, 73). For example, miR-7-5p inhibits the expression of ASD risk gene *PAX6* (74), a crucial transcription factor for neural tissue development, and regulates dopaminergic neuron differentiation (75). miR-7-5p also regulated the expression of ASD risk gene *SHANK3* and affected the dendritic spines in hippocampal neurons (76). Moreover, miR-7 could regulate cerebral cortex development through the p53 pathway (77). Our bioinformatic analysis shows that its related pathways include fatty acid biosynthesis, biotin metabolism, lysine degradation, and the Hippo signaling pathway (Supplementary Material 7, Figure 2B). Therefore, it is reasonable to speculate that miR-7 dysregulation may lead to neurologic disorders including ASD.

Each ASD-miRNA was validated to target several ASD risk genes and involves many signaling pathways (Supplementary Materials 7–9). *TNRC6B, PTEN, AGO1, AGO2, SKI*, and *SMAD4* are among the most commonly targeted ASD genes (Supplementary Materials 7–9). *TNRC6B, AGO1*, and *AGO2* participate in miRNA-mediated translational inhibition (78), which can affect a variety of cellular functions, given the various targets in this pathway. *PTEN* gene has been identified as a strong candidate gene for ASD (79). Defective *PTEN* protein interacts with tumor protein p53 to suppress energy production in neurons, causing detrimental changes in mitochondrial DNA and abnormal levels of energy production in the cerebellum and hippocampus, regions of the brain vital to social function and cognition, and Pten haploinsufficient mice exhibit autistic-like behavior with brain mitochondrial dysfunction and accumulation of mitochondrial DNA loss (80). Moreover, PTEN antagonizes PI3K-AKT signaling, which is essential for axon guidance and dendritic outgrowth (81). So *PTEN*-deficiency may affect neuronal connectivity, synaptic plasticity, and the development of autistic behavior (81). The SKI protein regulates the TGF-beta pathway by interacting with SMAD proteins, such as SMAD4, and plays a key role in tissue development (including the brain) during embryogenesis (82).

Tissue subgroup analysis showed that ASD-miRNAs in the brain, blood, and saliva participate in the FoxO signaling pathway (Figures 2A–C; Supplementary Materials 7–9), and several ASD risk genes are present in this pathway (Supplementary Materials 7–9). Recently, FoxO transcription factors have emerged as important regulators of cell development and function in the nervous system (83) and are implicated in neurological diseases such as Parkinson's disease and Huntington's disease (83, 84). A growing body of evidence demonstrates that miRNAs can directly regulate FoxO transcripts in various physiological and pathological conditions (85). However, there is still a lack of literature on the FoxO signaling pathway in ASD.

Notably, ASD-miRNAs were implicated in many cancer pathways (Supplementary Materials 7–9; Figures 2A–C). This supports evidence that ASD shares overlapping genes and pathways with cancers (86). For example, several ASD-miRNAs (miR-21-3p, miR-106a-5p, miR-155-5p, miR-92a-3p, miR-23a-3p, miR-106b-5p, and miR-19b-3p) have been shown to target *PTEN* (Supplementary Materials 7–9), a ubiquitously expressed tumor suppressor that is commonly inactivated in human cancers (87). Additionally, several ASD risk genes, including *PTEN*, are regulators of p53, which act as a tumor suppressor. Besides, the p53 tumor suppressor network cross-talks with the miRNA regulation system (88). For example, dysregulated microRNAs in ASD (miR-15a-5p, miR-19b-3p, and miR-92a-3p) target the p53 pathway (Supplementary Material 8; Figure 2A). In contrast, the p53 pathway could downregulate microRNAs such as let-7a and miR-17/92 cluster (miR-19b-1, miR-20a, and miR-92a) and upregulates microRNAs such as miR-107, miR-34c, miR-145, miR-15a, miR-23b, and miR-486 (89, 90). These miRNAs mediating p53 function in tumor suppression are also dysregulated in ASD. Evidence showed that the *p53* gene is more deficit in ASD children and their fathers (91). So dysregulated miRNAs and the p53 signaling pathway may impact each other mutually and contribute to ASD. Since ASD and cancer have mechanistic similarities, these have clinical importance, such as repurposing feasible cancer drugs for ASD-targeted therapies (86).

It should be highlighted that ASD-miRNAs are implicated in various metabolic pathways (Supplementary Materials 7–9; Figures 2A–C). This is consistent with the clinical finding that ASD persons have various metabolic problems (92–94). One explanation is mitochondria dysfunction since mitochondria are vital for bioenergetic and biosynthetic processes, including lipids, amino acids, and nucleotide metabolism (95). Many microRNAs including miR-23a-3p, miR-23b-3p, miR-107, and miR-103 are found in mitochondria (96); they may regulate various biological pathways, such as p53 signaling, neurotrophin signaling, TGF cycle, cell cycle, and ubiquitin-mediated proteolysis (97). For example, miR-23a-3p and miR-23b-3p bind to (glutaminase) GLS-mRNA and suppress its translation; thus, they are involved in the mitochondrial amino acid metabolism (96, 98). miR-107 and miR-103 regulate insulin signaling and glucose homeostasis (99). Cellular nutrient and energy-sensing by mTOR signaling regulates almost all aspects of metabolism and mitochondrial biogenesis and plays an important role in glucose homeostasis, lipid homeostasis, immune function, brain function, cancer, etc. (100, 101). Notably, several ASD risk genes are present in mTOR signaling, including *IGF1, MTOR, PIK3R2, PTEN, RHEB, TSC1*, and *TSC2*. Some of them (*PTEN* and *TSC2*) are direct target genes of p53, which regulates mTOR signaling and many metabolic activities (102). Taken together, microRNA, metabolism, and mitochondria are closely interconnected and associated with other cellular pathways, which may partially explain the etiology of mitochondrial dysfunction and diverse clinical manifestations of ASD.

It is well-known that immune dysregulation/inflammation participates in the pathogenesis of ASD. Reinforcing this perspective, our pathway enrichment analysis shows that ASD-miRNAs are involved in immune/inflammation-associated pathways, such as the NF-κB, Hippo, TGF-beta, and mTOR signaling pathways (Figures 2A–C; Supplementary Materials 7–9). The TGF-beta signaling pathway is a crucial regulator of the immune system and plays a critical role in the regulation of inflammation and embryo development (103). Evidence shows that TGF-beta1 regulates the PI3K/Akt/Wnt/beta-catenin signaling pathway and restores hippocampal synaptic plasticity and memory (104). TGF-beta1 also activates the MAPK signaling pathway and modulates neurogenesis (105). TGF-beta can also activate other pathways including mTOR signaling pathways and the NF-κB pathway (106). Moreover, TGF-beta signaling cross-talks with miRNAs and upregulates miRNAs such as the miR-181 family, the miR-17/92 cluster, miR-155, and the miR-23/24/27 cluster; downregulates the miR-200 family, miR-203, let-7, miR-34a, and miR-584 (107). On the other hand, miRNAs including the miR-106b/205 cluster (including miR-106b-5p and miR-93-5p) regulate TGF-beta signaling by convergently suppressing a range of TGF-beta signaling components, such as *SMAD4* (107). This may partly explain the reason why microRNAs are dysregulated in ASD. However, the functions of TGF-beta in ASD are little understood (108). Further research is needed to investigate the role of microRNAs and TGF-beta signaling in immune dysregulation in ASD.

The cadherin genes (including *PCDH10, CDH5, CDH8, CDH9*, and *CDH15*) are ASD risk genes and function as essential cell adhesion molecules. Some of them may interact with beta-catenin, which is encoded by the *CTNNB1* gene (a validated target of miR-155-5p, miR-27a-3p, and miR-106a-5p, etc.). Cadherins act as an intracellular signal transducer in various signaling pathways including the adherens junctions and the Hippo pathway and play a critical role in the development and cellular function (109).

Adherens junctions are cadherin-based protein complexes, which exist at intercellular adhesions of endothelial and epithelial tissues and play a critical role in embryogenesis and cortical development (110). Evidence confirmed the links between adherens junction and ASD (111). Interestingly, a functional local RNA interference system has been recently found in the epithelial adhesion junctions (112), which affects miRNAs and mRNAs, indicating that miRNAs play an important role in regulating epithelial and endothelial cell functions in development and disease (113). For example, miR-155 adversely affected brain–blood-barrier function during neuroinflammation by targeting cell–cell complex molecules, such as AA2, claudin-1, and molecules that are critical in cell-to-extracellular matrix (ECM) interactions including dedicator of cytokinesis 1 and syntenin-1 (114). Thus, miRNAs may contribute to adherens junctions dysfunction and brain–blood-barrier and intestinal epithelial barriers impairment in ASD (115, 116).

The extracellular matrix and its receptors are essential for neuronal migration during brain development and are involved in the maintenance of stable neuronal connections and regulation of synaptic plasticity (117). ASD risk genes including *RELN, LAMB1*, and *THBS1* are involved in ECM-receptor interaction. However, there is still a lack of literature on ECM-receptor interaction in ASD.

Adherens junctions, extracellular matrix, or cadherins could also regulate the Hippo pathway activity (118, 119), which plays a critical role in regulating development and organ size (120). Hippo signaling also cross-talks with innate immunity and regulates inflammation (121). Several ASD risk genes are implicated in Hippo signaling, including *CTNNB1, SMAD4*, and *PPP2R1B*. However, there is still little research in the literature regarding the Hippo signaling pathway in ASD.

Thyroid hormones are important regulators of development, growth, and metabolism and crucial for normal brain development. Thyroid hormones regulate AMPK activity and fatty acid metabolism in the central nervous system (122). Maternal thyroid disorders during pregnancy were found to be associated with an increased risk of ASD in offspring (123), and brain genes were altered (124). However, few studies have examined whether postnatal thyroid hormone levels influence the risk of ASD and the underlying mechanisms remain unclear (125). The thyroid hormone and its receptor-related genes (including *THRA, TPO, TRIP12*, and *NR4A2*) have been linked with ASD, and miRNAs are necessary for thyroid hormone production (126). Further studies are needed to investigate the microRNA regulation of thyroid hormones in ASD during pregnancy and postnatal.

Estrogen and estrogen receptors play critical roles in brain development and functions, including synaptogenesis, corticogenesis, cognition, and learning (127). Evidence showed that prenatal and postnatal estrogen signaling impairment may contribute to ASD (128, 129). Consistent with this, several ASD-miRNAs (including miR-142-3p, miR-151a-3p, miR-19b-3p, miR-32-5p, miR-221-3p, miR-320a, miR-338-5p, and miR-423-5p) target ASD risk genes including *PIK3R2, ADCY3, ITPR1*, and *GNAI1* (Supplementary Materials 7–9), which are also implicated in estrogen signaling.

Interestingly, the ASD-miRNAs target circadian rhythm genes including *RORA, RORB, PER1, PER2, BTRC, NPAS2, NR1D1*, and *CSNK1E*, which are also ASD risk genes (Supplementary Materials 7–9). Circadian rhythm disorders are associated with neurological (130) and other functional impairments in ASD (131). Evidence suggests that circadian rhythm genes may be correlated with ASD (132) and cause sleep disorders that are common in ASD (133). Moreover, salivary microRNAs (miR-24-3p, miR-200b-3p, miR-203a-3p, and miR-26a-5p) are associated with sleep disorders in children with ASD, so microRNAs in biofluids such as saliva could have diagnostic and therapeutic values in circadian rhythm disorder and ASD (134).

Collectively, this study clarified overlapping altered microRNAs in ASD and explored their target genes and associated pathways. We also highlight pathways that are significantly enriched while being less studied in ASD, such as adherens junctions, ECM receptor interaction, FoxO, Hippo, and TGF-beta signaling pathway.

Some limitations of this study exist. First, as microRNA profiling and analysis methods are heterogeneous among studies and much raw data are not available, it is difficult to perform a quantitative meta-analysis. Second, various diagnostic approaches were employed, and six studies did not report diagnostic tools (10, 14, 18, 33, 35, 53). The change of diagnostic criteria, for example, from DSM-IV to DSM-V, may lead to the inconsistency of research participants. The heterogeneity of diagnostic criteria and participants made comparisons between studies challenging. Subgroup analysis stratifying by homogeneous characteristics dimensionally or categorically within ASD would be helpful. However, this was not practicable as the included studies did not give sufficient phenotype/behavioral information on ASD individuals. It should also be noticed that some dysregulated microRNAs are located on the X chromosome and miR-29c-3p (27), miR-4732-5p, and miR-423-3p show gender differences (32). However, this link has not been identified in other studies (19, 22, 33, 38, 39). In addition, some research only included male or female ASD participants. Thus, it is unclear if particular miRNA dysregulation in ASD is gender-specific. Given the heterogeneity and gender differences in ASD, future microRNA expression research with more female participants and stratification by ASD subtype or gender will be valuable.

## Conclusion

In conclusion, our systematic review on miRNA expression profiling studies identified a number of altered microRNAs in ASD, especially miR-23a-3p, miR-27a-3p, miR-106b-5p, miR-93-5p, miR-7-5p, miR-146a-5p, and miR-155-5p. Some of these microRNAs have the potential to serve as biomarkers for ASD. Each of these altered microRNAs was validated to target many ASD risk genes. The target genes of these microRNAs are implicated in various pathways associated with ASD and form very rich networks, which further highlights the importance of these microRNAs in ASD etiology. However, few studies have reported the implication of microRNAs and some associated pathways in ASD pathogenesis, such as adherens junctions, ECM receptor interaction, FoxO, Hippo, TGF-beta signaling pathway, etc. More research is needed to examine microRNA expression in ASD and their associated target genes and pathways to better understand ASD pathogenesis. This may contribute to the use of microRNAs as potential personalized biomarkers and therapeutic targets.

## Data Availability Statement

The original contributions presented in the study are included in the article/Supplementary Material, further inquiries can be directed to the corresponding author.

## Author Contributions

Z-XH: conceptualization, methodology, software, formal analysis, bioinformatic analysis, investigation, resources, data extraction, data curation, writing - original draft, writing - review and editing, visualization, and funding acquisition (Qihang Funds of Fujian Medical University). YC: conceptualization, methodology, writing - review and editing, supervision, project administration, and funding acquisition. H-RG: data extraction and data curation. G-FC: data extraction. All authors read and approved the final manuscript.

## Funding

This research was supported by the National Key Research and Development Program of China (Grant No. 2016YFC1306204), the Joint Funds for the innovation of science and Technology, Fujian province (Grant No. 2017Y9043), and Qihang Funds of Fujian Medical University (Grant No. 2018QH2032). The funding sources had no role in the design and conduct of the study; collection, management, analysis, and interpretation of the data; preparation, review, or approval of the manuscript; and decision to submit the manuscript for publication.

## Conflict of Interest

The authors declare that the research was conducted in the absence of any commercial or financial relationships that could be construed as a potential conflict of interest.

## Publisher's Note

All claims expressed in this article are solely those of the authors and do not necessarily represent those of their affiliated organizations, or those of the publisher, the editors and the reviewers. Any product that may be evaluated in this article, or claim that may be made by its manufacturer, is not guaranteed or endorsed by the publisher.

## Abbreviations

ADCY3: adenylate cyclase 3
ADI-R: Autism Diagnostic Interview-Revised
ADOS: Autism Diagnostic Observation Schedule
AGO1: argonaute RISC component 1
Akt: also known as protein kinase B
AMPK: 5′ adenosine monophosphate-activated protein kinase
ASD: Autism spectrum disorder
BTRC: beta-transducin repeat containing E3 ubiquitin protein ligase
CARS: Childhood Autism Rating Scale
CARS-II, Childhood Autism Rating Scale: Second edition
CDH5: cadherin 5
CDH8: cadherin 8
CDH9: cadherin 9
CHD8: chromodomain helicase DNA binding protein 8
CSNK1E: casein kinase 1 epsilon
Ct: cycles-to-threshold
CTNNB1: catenin beta 1
CUL3: cullin 3
DSM-5, Diagnostic and Statistical Manual of Mental Disorders: 5th Edition
DSM-IV, Diagnostic and Statistical Manual of Mental Disorders: 4th Edition
DSM-IV-TR, Diagnostic and Statistical Manual of Mental Disorders, 4th Edition: Text Revision
ECM: extracellular matrix
FC: fold change
FDR: false discovery rate correction
FoxO: forkhead box O
GNAI1: G protein subunit alpha i1
IGF1: insulin like growth factor 1
IL6: interleukin 6
ITPR1, Inositol 1, 4: 5-trisphosphate receptor type 1
KDM3B: Lysine Demethylase 3B
KDM6B: lysine demethylase 6B
KEGG: Kyoto Encyclopedia of Genes and Genomes
KMT2A: lysine methyltransferase 2A
KMT2C: lysine methyltransferase 2C
KMT5B: lysine methyltransferase 5B
LAMB1: laminin subunit beta 1
MAPK: mitogen-activated protein kinase
MECP2: Methyl CpG binding protein 2
MED13: mediator complex subunit 13
mTOR: mammalian (mechanistic) target of rapamycin
MTOR: mechanistic target of rapamycin kinase
N/A: not available
NF-kappa B: Nuclear factor-kappa B
NPAS2: neuronal PAS domain protein 2
NR1D1: nuclear receptor subfamily 1 group D member 1
NR4A2: nuclear receptor subfamily 4 group A member 2
NSD1: nuclear receptor binding SET domain protein 1
PAX6: paired box 6
PBMC: peripheral blood monocytes
PCDH10: protocadherin 10
PER1: period circadian regulator 1
PER2: period circadian regulator 2
PI3K: phosphatidylinositol 3′ -kinase
PIK3R2: phosphoinositide-3-kinase regulatory subunit 2
PPP2R1B: protein phosphatase 2 scaffold subunit Abeta
PRISMA: Preferred Reporting Items for Systematic reviews and Meta-Analyses
PTEN: phosphatase and tensin homolog
qRT-PCR: quantitative reverse-transcription/real time polymerase chain reaction
RELN: reelin
RHEB, Ras homolog: mTORC1binding
RNA-Seq: ribonucleic acid sequencing
RORA: RAR related orphan receptor A
RORB: RAR related orphan receptor B
SETD1B: SET domain containing 1B
SHANK3: SH3 and multiple ankyrin repeat domains 3
SKI: Sloan–Kettering Institute proto-oncogene
SMAD: mothers against decapentaplegic homolog
TBL1XR1: transducin beta like 1 X-linked receptor 1
TCF4: transcription factor 4
TGF: transforming growth factor
THBS1: thrombospondin 1
THRA: thyroid hormone receptor alpha
TNRC6B: trinucleotide repeat containing adaptor 6B
TPO: thyroid peroxidase
TRIP12: thyroid hormone receptor interactor 12
TSC1: tuberous sclerosis complex 1
TSC2: tuberous sclerosis complex 2
Wnt: Wingless-type/Int-1.
