# Supplementary material for: Systematic Review and Bioinformatic Analysis of microRNA Expression in Autism Spectrum Disorder Identifies Pathways Associated With Cancer, Metabolism, Cell Signaling, and Cell Adhesion
Source: Front Psychiatry. 2021 Oct 21;12:630876. doi: 10.3389/fpsyt.2021.630876 (PMC8566729; doi:10.3389/fpsyt.2021.630876)
Supplement: Supplementary file 1 [file Table_1.docx]

**Search criteria**

**Date of search: May 11th 2020**

**Search strategy**

**Pubmed**

#1

(("Autism Spectrum Disorder"[Mesh]) OR ("Autistic Disorder"[Mesh])) OR (((((((((((((((((((((((((((((("autism spectrum disorder"[Title/Abstract]) OR ("spectrum disorders, autism"[Title/Abstract])) OR ("autism spectrum disorders"[Title/Abstract])) OR ("autistic disorder"[Title/Abstract])) OR ("disorder, autistic"[Title/Abstract])) OR ("disorders, autistic"[Title/Abstract])) OR ("kanner's syndrome"[Title/Abstract])) OR ("kanner syndrome"[Title/Abstract])) OR ("kanner s syndrome"[Title/Abstract])) OR ("autism, infantile"[Title/Abstract])) OR ("infantile autism"[Title/Abstract])) OR ("autism, early infantile"[Title/Abstract])) OR ("early infantile autism"[Title/Abstract])) OR ("infantile autism, early"[Title/Abstract])) OR ("Child Development Disorders, Pervasive"[Mesh]))) OR ("pervasive child development disorders"[Title/Abstract])) OR ("pervasive development disorders"[Title/Abstract])) OR ("Developmental Disabilities"[Mesh])) OR ("pervasive next development* next disorder*"[Title/Abstract])) OR ("pervasive near/3 child*"[Title/Abstract])) OR (PDD[Title/Abstract])) OR (pdds[Title/Abstract])) OR (pdd-nos[Title/Abstract])) OR (ASD[Title/Abstract])) OR (asds[Title/Abstract])) OR (autis*[Title/Abstract])) OR (asperger*[Title/Abstract])) OR (kanner*[Title/Abstract])) OR ("childhood schizophrenia"[Title/Abstract])) OR (Rett*[Title/Abstract]))

#2

((((miR* OR microRNA* OR miRNA*)) OR ((((((((((((((((((((microrna) OR mirnas) OR micro rna) OR rna, micro) OR mirna) OR primary microrna) OR microrna, primary) OR primary mirna) OR mirna, primary) OR pri-mir small temporal) OR temporal rna, small) OR sirna) OR pre mirna) OR pre-mirna) OR small temporal rna)) OR "MicroRNAs"[Mesh]))))))

#3

#1 AND #2

**EMBASE**

#1

'rett*':ab,kw,ti OR 'childhood schizophrenia':ab,kw,ti OR 'kanner*':ab,kw,ti OR 'asperger*':ab,kw,ti OR 'autis*':ab,kw,ti OR 'asds':ab,kw,ti OR 'asd':ab,kw,ti OR 'pdd‐nos':ab,kw,ti OR 'pdds':ab,kw,ti OR 'pdd':ab,kw,ti OR 'pervasive near/ child*':ab,kw,ti OR 'pervasive next development* next disorder*':ab,kw,ti OR 'developmental disorder'/exp OR 'developmental disorder' OR 'pervasive development disorders':ab,kw,ti OR 'pervasive child development disorders':ab,kw,ti OR 'child development disorders pervasive':ab,kw,ti OR 'infantile autism early':ab,kw,ti OR 'early infantile autism':ab,kw,ti OR 'autism early infantile':ab,kw,ti OR 'infantile autism':ab,kw,ti OR 'autism infantile':ab,kw,ti OR 'kanner syndrome':ab,kw,ti OR 'kanner* syndrome':ab,kw,ti OR 'kanners syndrome':ab,kw,ti OR 'disorders autistic':ab,kw,ti OR 'disorder autistic':ab,kw,ti OR 'autistic disorder':ab,kw,ti OR 'autism spectrum disorders':ab,kw,ti OR 'spectrum disorders autism':ab,kw,ti OR 'autism':ab,kw,ti OR 'autism'/exp

#2

'microrna'/exp

153,471

#3

'microrna*':ab,kw,ti OR 'mirna*':ab,kw,ti OR 'pre mirna':ab,kw,ti OR 'pre-mirna':ab,kw,ti OR 'small temporal rna':ab,kw,ti OR 'strna':ab,kw,ti OR 'temporal rna, small':ab,kw,ti OR 'rna, small temporal':ab,kw,ti OR 'pri mirna':ab,kw,ti OR 'pri-mirna':ab,kw,ti OR 'mirna, primary':ab,kw,ti OR 'primary mirna':ab,kw,ti OR 'microrna, primary':ab,kw,ti OR 'primary microrna':ab,kw,ti OR 'mirna':ab,kw,ti OR 'rna, micro':ab,kw,ti OR 'micro rna':ab,kw,ti OR 'mirnas':ab,kw,ti OR 'microrna':ab,kw,ti OR 'micrornas':ab,kw,ti

134,750

#4

#2 OR #3

162,283

#5

#1 AND #4

716

**OVID**

**Database: Search All Journals@Ovid, FSU Books@Ovid, FSU Full-Text Journals@Ovid, Ovid MEDLINE ALL**

Search Strategy:

1 (Autism Spectrum Disorder or Spectrum Disorders Autism or Autism Spectrum Disorders or Autistic Disorder or Disorder Autistic or Disorders Autistic or Kanner Syndrome or Kanner Syndrome or Kanners Syndrome or Autism Infantile or Autism Infantile or Autism or Autism Early Infantile or Early Infantile Autism or Infantile Autism or Child Development Disorders Pervasive or Pervasive Child Development Disorders or Pervasive Development Disorders or Developmental Disabilities or PDD or autis* or asperger* or kanner* or "childhood schizophrenia" or Rett* or PDDs or PDD-NOS or ASD or ASDs).ab,at,hw,kf,kw,sh. (104131)

2 ("MicroRNAs" or "MicroRNA" or "miRNAs" or "Micro RNA" or "RNA, Micro" or "miRNA" or "Primary MicroRNA" or "MicroRNA, Primary" or "Primary miRNA" or "miRNA, Primary" or "pri-miRNA" or "pri miRNA" or "RNA, Small Temporal" or "stRNA" or "Small Temporal RNA" or "pre-miRNA" or "mirna* " or "microrna* ").af. (196942)

3 1 and 2 (668)

**Web of Science Core Collection (1900-present):**

**Science Citation Index Expanded (SCI-EXPANDED) --1900-present**

**Social Sciences Citation Index (SSCI) --1900-present**

**Arts & Humanities Citation Index (A&HCI) --1975-present**

**Emerging Sources Citation Index (ESCI) --2015-present**

# 1

TS=(“Autism Spectrum Disorder” OR “Spectrum Disorders, Autism” OR “Autism Spectrum Disorders” OR “Autistic Disorder” OR “Disorder, Autistic” OR “Disorders, Autistic” OR “Kanner's Syndrome” OR “Kanner Syndrome” OR “Kanners Syndrome” OR “Autism, Infantile” OR “Infantile Autism” OR Autism OR “Autism, Early Infantile” OR “Early Infantile Autism” OR “Infantile Autism, Early” OR "Child Development Disorders, Pervasive" OR “Pervasive Child Development Disorders” OR “Pervasive Development Disorders” OR "Child Development Disorders, Pervasive" OR "Developmental Disabilities" OR “pervasive next development* next disorder*” OR “pervasive near/3 child*” OR PDD or PDDs or PDD*NOS or ASD or ASDs OR autis* OR asperger* OR kanner* OR "childhood schizophrenia" OR Rett*)

# 2

TS=(MicroRNAs OR MicroRNA OR miRNAs OR "Micro RNA" OR "RNA, Micro" OR miRNA OR "Primary MicroRNA" OR "MicroRNA, Primary" OR "Primary miRNA" OR "miRNA, Primary" OR "pri-miRNA" OR "pri miRNA" OR "RNA, Small Temporal" OR "Temporal RNA, Small" OR stRNA OR "Small Temporal RNA" OR pre-miRNA OR "pre miRNA" OR microRNA* OR miRNA*)

# 3

#1 AND #2

**Scopus**

( ( ( ( ( ( TITLE-ABS-KEY ( "Autism Spectrum Disorder" ) OR TITLE-ABS-KEY ( "Autism Spectrum Disorders" ) OR TITLE-ABS-KEY ( "Spectrum Disorders, Autism" ) OR TITLE-ABS-KEY ( "Autistic Disorder" ) OR TITLE-ABS-KEY ( "Autistic Disorder" ) OR TITLE-ABS-KEY ( "Disorder, Autistic" ) OR TITLE-ABS-KEY ( "Disorders, Autistic" ) OR TITLE-ABS-KEY ( "Kanner's Syndrome" ) OR TITLE-ABS-KEY ( "Kanner Syndrome" ) OR TITLE-ABS-KEY ( "Kanners Syndrome" ) OR TITLE-ABS-KEY ( "Autism, Infantile" ) OR TITLE-ABS-KEY ( "Infantile Autism" ) OR TITLE-ABS-KEY ( "Autism, Early Infantile" ) OR TITLE-ABS-KEY ( "Early Infantile Autism" ) OR TITLE-ABS-KEY ( "Infantile Autism, Early" ) OR TITLE-ABS-KEY ( "Child Development Disorders, Pervasive" ) OR TITLE-ABS-KEY ( "Pervasive Child Development Disorders" ) OR TITLE-ABS-KEY ( "Pervasive Development Disorders" ) OR TITLE-ABS-KEY ( "Developmental Disabilities" ) OR TITLE-ABS-KEY ( "pervasive next development* next disorder*" ) OR TITLE-ABS-KEY ( "pervasive near/3 child*" ) OR TITLE-ABS-KEY ( "PDD" ) OR TITLE-ABS-KEY ( "PDDs" ) OR TITLE-ABS-KEY ( "PDD‐NOS" ) OR TITLE-ABS-KEY ( "ASD" ) OR TITLE-ABS-KEY ( "ASDs" ) OR TITLE-ABS-KEY ( "autis*" ) OR TITLE-ABS-KEY ( "asperger*" ) OR TITLE-ABS-KEY ( "kanner*" ) OR TITLE-ABS-KEY ( "childhood schizophrenia" ) OR TITLE-ABS-KEY ( "Rett*" ) ) ) ) ) ) ) AND ( ( ( ( ( ( TITLE-ABS-KEY ( "MicroRNAs" ) OR TITLE-ABS-KEY ( "MicroRNA" ) OR TITLE-ABS-KEY ( "miRNAs" ) OR TITLE-ABS-KEY ( "Micro RNA" ) OR TITLE-ABS-KEY ( "RNA, Micro" ) OR TITLE-ABS-KEY ( "miRNA" ) OR TITLE-ABS-KEY ( "Primary MicroRNA" ) OR TITLE-ABS-KEY ( "MicroRNA, Primary" ) OR TITLE-ABS-KEY ( "Primary miRNA" ) OR TITLE-ABS-KEY ( "miRNA, Primary" ) OR TITLE-ABS-KEY ( "pri-miRNA" ) OR TITLE-ABS-KEY ( "pri miRNA" ) OR TITLE-ABS-KEY ( "RNA, Small Temporal" ) OR TITLE-ABS-KEY ( "Temporal RNA, Small" ) OR TITLE-ABS-KEY ( "stRNA" ) OR TITLE-ABS-KEY ( "Small Temporal RNA" ) OR TITLE-ABS-KEY ( "pre-miRNA" ) OR TITLE-ABS-KEY ( "pre miRNA" ) OR TITLE-ABS-KEY ( "mirna*" ) OR TITLE-ABS-KEY ( "microrna*" ) OR TITLE-ABS-KEY ( "miRNA*" ) OR TITLE-ABS-KEY ( "MicroRNA*" ) OR TITLE-ABS-KEY ( "miRNA*" ) ) ) ) ) ) )

**#hits per database:**

PUBMED: 432

EMBASE: 716

OVID: 668

Web of science: 356

SCOPUS: 546

Total: 2718 potentially relevant references identified with **at least** 1385 duplicate references
